# Supplementary material for: Patterns of Intron Gain and Loss in Fungi
Source: PLoS Biol. 2004 Nov 30;2(12):e422. doi: 10.1371/journal.pbio.0020422 (PMC532390; doi:10.1371/journal.pbio.0020422)
Supplement: Table S1 — Also available at http://genes.mit.edu/NielsenEtAl/. (4.3 MB ZIP). [file pbio.0020422.st001.zip › NielsenEtAl/html/524.html]

AN8194.1.NCU03800.1.MG07471.1.FG06115.1


```
 CLUSTAL W (1.82) Multiple Sequence Alignments - Introns Inserted


Sequence 1: MG07471.1	220 aa
Sequence 2: FG06115.1	210 aa
Sequence 3: NCU03800.1	221 aa
Sequence 4: AN8194.1	210 aa
Alignment Length: 222 aa
Number Identitical Residues: 111 aa
Alignment Score (without introns) 5272


MG07471.1 	MQLLLFTYGLFAWLAAQAAATALTYKLHASEKACFYTAGKTKGEKIAFYFA0VQSGGSFD
NCU03800.1	MKLSTITCALLG-LASEVAATALTYKLVANEKACFFATTKKANEKIAFYFA0VQSGGSFD
FG06115.1 	MRLPTLVAGLFACLATNVAATALTYKLDAHEKACFHTTTKKQGEKIAFYFA0VQSGGSFD
AN8194.1  	MRFTALLIGLLAYLVSSVTATALTYRLEANEKACFYKFVEQRNTKVAFYFA0VQSGGSFD
          	*::  :  .*:. *.:..:******:* * *****.   :  . *:***** ********

MG07471.1 	VDYEVIGPTGKVILDGEAERQGDFVFTVNDIGEYRFCFSNTMSSYDDKVIDFEIA0VENE
NCU03800.1	IDYEVTGPDGNVIMDGQKERQGDFVFTGKQIGDYKFCFNNEMSTFAEKFVDFELA0VEDE
FG06115.1 	VDYVVEGPNGKIIMDGQKERQGDFVFSANVVGDYSFCFDNEMSTFAEKYVDFEIA~VENE
AN8194.1  	VDYAVVGPGEKPVLDGTKERQGDFVFTAQSVGEYRFCFNNEMSTFAEKMVDFEIA0LENE
          	:** * **  : ::**  ********: : :*:* ***.* **:: :* :***:* :*:*

MG07471.1 	ARVGLPSKKGTSPEQTTTLEESAFKISGQLSSITRHQKYFRTRENRNFATVRSTEKRIIN
NCU03800.1	ARVTIPSKQGSSPEQTSALEESLFKISMQLSTVTRNQKYFRTRENRNFSTVRSTEKRIVN
FG06115.1 	SRAQLPSKQGTTPEQTSALEESIFKISGQLSTISRNQKYFRTRENRNFATVNSTEGRIIN
AN8194.1  	SRTELPSRQGANPEQASAIEESIFKLSAQLSTISRNQKYFRTRENRNFSTVRSTERRIWN
          	:*. :**::*:.***::::*** **:* ***:::*:************:**.*** ** *

MG07471.1 	FSLIQCGLIVLMGALQVFIVRFFFQ0TTCAA~VMERNSRSLLGE--
NCU03800.1	FSLIQILLVIAMGALQVFIVRFFFQ0AGVRM~LIGWTTQGARKGYV
FG06115.1 	FSMIQIGLIICMGALQVFVVRFFFQ0GARKG~YV------------
AN8194.1  	FSIIESLMMVSMAGLQVFIVRFFFQ~GARKG1YV------------
          	**:*:  ::: *..****:******        :
```
